# Is Nuclear Factor Erythroid 2-Related Factor 2 a Target for the Intervention of Cytokine Storms?

**DOI:** 10.3390/antiox12010172

**Published:** 2023-01-11

**Authors:** Zihang Liu, Panpan Deng, Shengnan Liu, Yiying Bian, Yuanyuan Xu, Qiang Zhang, Huihui Wang, Jingbo Pi

**Affiliations:** 1 The First Department of Clinical Medicine, China Medical University, Shenyang 110122, China; 2 Program of Environmental Toxicology, School of Public Health, China Medical University, Shenyang 110122, China; 3 Group of Chronic Disease and Environmental Genomics, School of Public Health, China Medical University, Shenyang 110122, China; 4 Gangarosa Department of Environmental Health, Rollins School of Public Health, Emory University, Atlanta, GA 30322, USA

**Keywords:** NRF2, cytokine storm, cytokine, oxidative stress, inflammation

## Abstract

The term “cytokine storm” describes an acute pathophysiologic state of the immune system characterized by a burst of cytokine release, systemic inflammatory response, and multiple organ failure, which are crucial determinants of many disease outcomes. In light of the complexity of cytokine storms, specific strategies are needed to prevent and alleviate their occurrence and deterioration. Nuclear factor erythroid 2-related factor 2 (NRF2) is a CNC-basic region-leucine zipper protein that serves as a master transcription factor in maintaining cellular redox homeostasis by orchestrating the expression of many antioxidant and phase II detoxification enzymes. Given that inflammatory response is intertwined with oxidative stress, it is reasonable to assume that NRF2 activation limits inflammation and thus cytokine storms. As NRF2 can mitigate inflammation at many levels, it has emerged as a potential target to prevent and treat cytokine storms. In this review, we summarized the cytokine storms caused by different etiologies and the rationale of interventions, focusing mainly on NRF2 as a potential therapeutic target.

## 1. Introduction

The cytokine storm was first described in medical literature in 1993 and recognized later as a series of exaggerated immune responses characterized by a burst of pro-inflammatory cytokine release, which usually causes acute systemic inflammatory response [1]. While there is still no widely accepted definition for cytokine storm, three key characteristics, including elevated circulating cytokines, acute systemic inflammation, and secondary multi-organ dysfunction, have been proposed as the identification criteria [2]. A cytokine storm is generally divided into the following three stages: primary loci stage, systemic inflammation, and multi-organ failure [2]. Although multiple general recommendations on anti-inflammatory treatments have been proposed for cytokine storms associated with a variety of diseases, including malignancy, rheumatologic disease, sepsis syndrome, and severe coronavirus disease 2019 (COVID-19) [2,3,4], there is still an urgent need to find novel strategies and methods to prevent and alleviate cytokine storms.

Nuclear factor erythroid 2-related factor 2 (NRF2) is a CNC-basic region-leucine zipper (CNC-bZIP) protein that functions as a key transcription factor regulating the cellular response against oxidative and electrophilic stresses. Kelch-like ECH-associated protein 1 (KEAP1), a substrate adaptor for the Cullin 3 (Cul3)-based E3 ubiquitin ligase complex, acts as a stress sensor and negative regulator for NRF2 stability [5]. Under unstressed conditions, KEAP1 sequesters NRF2 and mediates its degradation, which maintains NRF2 at low levels [6]. In response to oxidative and/or electrophilic challenge, the key cysteine residues on the KEAP1 could be oxidized, leading to conformation change and subsequently losing its function as a substrate adaptor for ubiquitin ligase, facilitating the escape of NRF2 from ubiquitination and nuclear accumulation. In the nucleus, NRF2 may form a heterodimer with small musculoaponeurotic fibrosarcoma (sMaf) proteins and bind to the antioxidant response elements (AREs), resulting in the transcription of a battery of target genes, which encode antioxidants, phase II detoxification enzymes, drug transporters and key molecules controlling cell metabolism [7,8,9]. In addition, accumulating evidence has shown that activation of the NRF2-ARE signaling pathway is crucial to the alleviation of inflammation. On the one hand, NRF2-induced cytoprotective proteins are likely to be important in the regulation of innate immune responses by repressing the expression of pro-inflammatory genes and potentiating the anti-inflammatory signaling [10]. On the other hand, NRF2-mediated antioxidant response might mitigate the activation of nuclear factor kappa-light chain-enhancer of activated B (NF-κB) to reduce the inflammatory response [11]. The absence of NRF2 exacerbates NF-κB activity, leading to increased cytokine production, whereas NF-κB, in turn, can modulate NRF2 transcription and activity [12]. In addition, NRF2 has also been found to regulate the transcription of a variety of cytokines in distinct ways.

Although the anti-inflammatory effects of NRF2 have been mainly attributed to its regulation in antioxidative genes, the molecular details underlying the effects are only partially characterized [10,13,14,15]. In light of the successful applications of multiple NRF2 activators in treating inflammation-related disorders, the effects of NRF2 activation against cytokine storms have been actively investigated. The present review provides an overview of the cytokine storms caused by different etiologies and the rationale of interventions, focusing particularly on NRF2 as a potential therapeutic target.

## 2. Cytokine Storms

Cytokine storms result from the collapse of complex immune networks, of which the key point is the disparity in forces between the pro-inflammatory and anti-inflammatory systems [2,16,17]. Cytokine storms can be divided into the following four types according to their initiating factors: pathogen, clinical treatment, autoimmune, and others [17,18,19,20]. In the case of pathogen-induced cytokine storms, disseminated bacteria or virus infections inducing sepsis and abnormally mobilized host immune system are evident [21,22]. A fraction of COVID-19 patients suffered from a life-threatening systemic inflammatory state, which was first described in children and adolescents, was termed as multisystem inflammatory syndrome [23]. In addition, a similar condition in adults has also been reported as multisystem inflammatory syndrome [24]. In COVID-19 patients, plasma levels of pro-inflammatory cytokines, including interleukin (IL)-1α, IL-1β, and tumor necrosis factor α (TNF-α), are elevated, compared to healthy adults [25,26]. In line with the findings in COVID-19 patients, induction of type I and III interferons (IFN), which are considered as the first line of cellular antiviral defenses, was observed to be delayed in the severe acute respiratory syndrome coronavirus 2 (SARS-CoV-2), resulting in a subsequent increase of IFN-γ and TNF-α, which trigger inflammatory cell death, tissue damage, and mortality in SARS-CoV-2 [21,27]. In addition, sepsis patients also show higher plasma levels of pro-inflammatory cytokines, including IL-6, IFN-γ, and TNF-α [28]. In contrast to pathogen-induced cytokine storms, treatment-induced cytokine storms are increasingly familiar to clinicians as more immune-cell engaging immunotherapies are used in clinics. For example, the cytokines released by chimeric antigen receptor (CAR) T-cells or macrophages activated in CAR T-cell therapy are the initiators of treatment-induced cytokine storms [29]. In the patients with CAR T-cell therapy, the serum levels of pro-inflammatory cytokines, including IL-6 and IFN-γ, elevated with the onset of cytokine storms [30]. In agreement with the effects of CAR T-cell therapy, cytokine storms could also happen in some patients with autoimmune disorders, such as familial Mediterranean fever and hemophagocytic lymphohistiocytosis. In these patients, innate cells abnormally release TNF-α, IFN-γ, IL-1β, and IL-6, and trigger cytokine storms [2,31,32,33]. Some non-infectious diseases, such as acute pancreatitis, may also induce cytokine storms [34]. These non-infectious diseases are not caused by specific pathogens but by some physical or chemical factors, which could trigger a series of excessive immune responses, leading to cytokine storms. In acute pancreatitis patients, high serum or plasma levels of IL-6 and IL-8 were observed in severe versus non-severe cases [35]. In general, cytokine storms are associated with immune disorders when the immune system of the host is mobilized toward a higher level. In contrast, regulatory T-cells (Tregs) can reduce the primary inflammatory responses and the tissue damage caused by systemic inflammation [36,37]. It is worth noting that the increase in the negative regulatory factors of inflammatory responses might be related to a period of low immune response called immunoparalysis, which increases the risk of sepsis and organ failure after a cytokine storm and leads to a poor prognosis [36,38].

**Figure 1:**
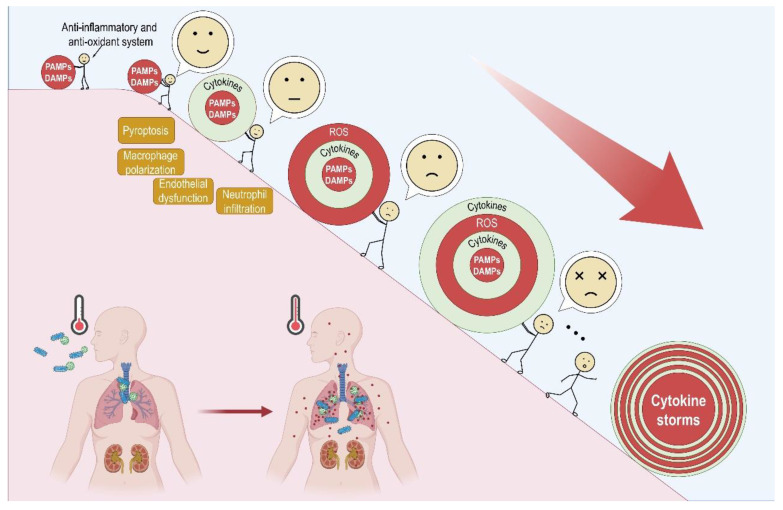
Schematic illustration of pathogen-induced cytokine storms. The PAMPs and DAMPs derived from PAMPs–induced cell damages are the primary initiators of cytokine storms, in which multiple key cellular events are involved. Without proper antioxidant and/or anti-inflammatory response, accumulating ROS and cytokines may form a positive feedback loop to aggravate inflammatory response at the infection loci, leading to a pathological burst of pro-inflammatory cytokines into the systemic circulation and multi-organ failure.

In pathogen-induced cytokine storms, as illustrated in Figure 1, the pathogen-associated molecular patterns (PAMPs) derived from various microorganisms, such as influenza A virus (IAV), SARS-CoV-2, and *Staphylococcus aureus*, might induce abnormal activation of primary inflammatory responses at the affected loci. The PAMPs that induce cytokine storms include RNA from viruses and superantigens from bacteria [39,40,41]. In the process, the characteristics of the PAMPs are crucial [42,43], as the infected cells may release a variety of damage-associated molecular patterns (DAMPs) in a PAMPs-dependent manner. In the case of virus infection, pattern recognition receptors (PRRs) sense PAMPs, which are often aberrant RNA structures formed during virus replication [44]. For example, the RNA of SARS-CoV-19 can stimulate membrane-bound PRR, Toll-like receptor (TLR)-3, 7, and cytosolic PRR, melanoma differentiation-associated gene 5, and lead to the release of cytokines, including IL-6 and TNF [39]. The DAMPs, such as reactive oxygen species (ROS) and cytokines, may form a positive feedback loop to elicit the primary acute inflammatory effects, leading to a pathological burst of pro-inflammatory cytokines with mismatched secretion of anti-inflammatory cytokines into the systemic circulation [45].

The primary acute inflammatory responses at the infection loci may develop into systemic inflammatory outcomes when ROS, cytokines, and other DAMPs are poured into the systemic circulation and spread throughout the body [16,17,46]. The systemic inflammatory effects, commonly observed in patients with cytokine storms, are fever, coagulopathies, changes in the fibrinolysis system and vascular tension, endothelial dysfunction, and anasarca. These changes are generally associated with increased levels of TNF-α, IFN-γ, IL-1β, and IL-6 in the systemic circulation [2,47]. In addition, the systemic inflammatory effects cause secondary damage to the primary lesion loci and distant tissues, including the vascular and lymphatic systems, central or peripheral nervous system, gastrointestinal system, lung, heart, liver, kidney, and skin. While TNF-α, IL-1β, and IL-6 are the most important cytokines in this damaging process [21,48,49,50], in the later phase of critical infectious disorders, such as severe COVID-19 patients, the systemic levels of IL-4, IL-10, and IL-13 may also increase [16,38].

The key cellular events in cytokine storms, including cell proliferation, polarization, differentiation, maturation, apoptosis, and cytokine release, are modulated by multiple signaling pathways. For example, in pathogen-induced cytokine storms, PAMP-induced DAMPs are recognized by PRRs (e.g., TLRs or nucleotide-binding oligomerization domain-containing protein 2 (NOD2)) that trigger pro-inflammatory signaling. It has been well documented that mitogen-activated protein kinase (MAPK)-extracellular signal-regulated kinase (ERK) and NF-κB pathways are commonly involved in inflammatory processes [51,52,53]. These pathways lead the primary immune cells toward inflammatory responses and control the expression of cytokines and other inflammatory mediators. As a result, the activated primary immune cells release aberrantly high levels of cytokines into the systemic circulation. For instance, virus replication is normally first detected by PRRs and induces transcriptional activation of IFN-III and IFN-I mediated by NF-κB and interferon regulator factors, respectively [54,55]. Next, upregulation of IFN-stimulated genes and recruitment and coordination of specific subsets of leukocytes is triggered. Interestingly, when SARS-CoV-2 first replicates at a low level, the IFN-dependent pathways are suppressed, which could lead to antivirus defense failure. In contrast, high titers of SARS-CoV-2 induce a strong IFN-dependent response, elevated chemokines and high expression of IL-6 [27]. In addition, Janus kinase-signal transducer and activators of transcription (JAK-STAT), MAPK-ERK, and NF-κB pathways are also involved in the downstream signal transduction of cytokines in target cells [56,57].

In summary, cytokine storm is a state of auto-amplifying cytokine production due to dysregulated host immune response to different triggers, including severe infections, diseases and immunotherapies. The excessive activation of immune cells and uncontrolled generation of pro-inflammatory cytokines may further result in systemic inflammation across multiple systems, leading to organ dysfunction and failure if not treated adequately.

## 3. Crosstalk between Oxidative Stress and Inflammatory Response

Oxidative stress is commonly described as a persistent imbalance between ROS production and its breakdown by distinct antioxidant systems in favor of oxidation, leading to disruption of redox signaling and/or oxidative damage [58,59,60]. In general, overproduced superoxide anion (O_2_^•−^) and nitric oxide (NO) and their derivatives, including hydrogen peroxide (H_2_O_2_), hydroxyl radical (HO^•−^), and peroxynitrite (ONOO^•−^) generated from pathophysiologic processes and subsequent free radical chain reactions, are the primary initiating factors to induce oxidative stress.

ROS can be produced at many sites via multiple enzymes, including mitochondria, NADPH oxidases (NOX), and NO synthetases [61,62,63,64]. While ROS are crucial to eliminate pathogens, they may also cause damage to local tissues and trigger various immune events. In infection-induced cytokine storms, the PAMPs derived from pathogens might cause damage to parenchymal cells via PRRs or even induce regulated cell death (RCD), which may generate and release more DAMPs [65,66,67]. ROS, on the one hand, may stimulate the expression and secretion of many cytokines, such as IL-1β, TNF-α, and IL-6. In turn, increased cytokines promote the activation of NOX, leading to more ROS production, forming a vicious cycle; on the other hand, ROS could also function as a signaling molecule to initiate the activation of NRF2-mediated antioxidant response and thus stop the ROS-cytokine loop. To understand the details involving the paradoxical roles of ROS in inflammatory response, we conducted a series of computational modeling studies. Our mathematical models showed that the concentration effect of NRF2 activation induced by ROS, such as H_2_O_2_, displays a nonlinear relationship [8,68]. When H_2_O_2_ is at physiologically relevant low levels, it cannot induce oxidation of KEAP1 and subsequent NRF2 activation. With the increase of H_2_O_2_ levels under certain stresses and/or pathogenic conditions, H_2_O_2_ could reach levels that could activate the KEAP1-NRF2-ARE system in a concentration-dependent manner. However, following further increase of H_2_O_2_, the steady-state concentration of NRF2 will lose its ultrasensitivity, and then enter the plateau stage, at which further increased H_2_O_2_ cannot effectively increase nuclear NRF2 levels. Thus, it is evident that the distinct paradoxical roles of ROS in inflammatory response are concentration dependent.

RCD, including pyroptosis, ferroptosis, and apoptosis, involves tightly structured signaling cascades and molecularly defined effector mechanisms, in which ROS play an important part [69,70]. A growing number of novel forms of RCD have been identified and known as crucial factors in various pathophysiological settings. Among the key molecules regulating RCD, the nucleotide-binding domain, leucine-rich-repeat-containing family, pyrin domain-containing 3 (NLRP3) inflammasome is noticeable, because it is widely involved in a variety of pathogenic conditions [51,71]. In cells exposed to PAMPs and/or DAMPs, the NLRP3 inflammasome detects microbial products and stressors and subsequently activates caspase-1, 4, 5, and 11, leading to the overproduction of IL-1β, IL-18, and other DAMPs [72]. It has been well documented that the activation of NLRP3 is critical in pyroptosis and is deeply influenced by ROS [73,74,75]. In addition, ROS also serve as key activating factors in ferroptosis, a distinct RCD caused by the accumulation of lipid peroxides through iron-mediated lipid peroxidation [69,76], and apoptosis, which involves the genetically determined elimination of cells initiated by caspase-8 and 9 and executed by caspase-3, 6, and 7 [77]. Thus, overproduction of ROS is critically important in a variety of RCD, which may aggravate the release of DAMPs, leading to a pro-inflammatory response.

ROS can function as signaling molecules to initiate the primary inflammatory responses and the secondary damage in cytokine storms [78,79]. Increased levels of ROS initiate and maintain the abnormal activation of NF-κB and MAPK and the excess of pro-inflammatory cytokines, such as IL-6 [80,81,82]. IL-6, as a key DAMP produced by PAMPs- and/or DAMPs-activated macrophages, might bind with two different receptors, bringing about diametrically opposite effects [83]. On the one hand, IL-6 mostly drives the acute-phase response by binding to the soluble receptor and subsequently activating a variety of immune cells including T-cells. In turn, activated T-cells and natural killer (NK) cells produce IFN-γ and augment macrophage activation, constituting a positive feedback loop among the immune cells to sustain the inflammation environment and promote anti-pathogen immunity [84]. On the other hand, IL-6 may also trigger an anti-inflammatory response via binding to the membrane-bound IL-6 receptor (mIL-6R) [45,85]. In line with the critical roles of IL-6 in inflammation, TNF-α may stimulate mitochondrial ROS production and trigger inflammatory cell death, tissue damage, and mortality via binding to the membrane-bound or soluble receptors [86], and thus accelerate the ROS–cytokines vicious cycle.

The molecular details underlying the stimulatory effects of ROS on cytokine production are complicated. The ROS produced from NOX on the membrane interact directly with the canonical pathways by affecting the inhibitor of NF-κB (IκB) and IκB kinase (IKK) β or the activation of NF-κB-inducing kinase, the upstream kinase in the alternative pathway [87,88]. In addition, ROS can activate MAPK kinase and initiate the MAPK cascade [89,90]. Activation of the MAPKs leads to phosphorylation and activation of p38 present in the cytoplasm or nucleus, which initiates the inflammatory response. MAPKs are a family of protein kinases that respond to diverse stimuli by inducing pro-inflammatory cytokines (e.g., IL-1β, TNF-α, and IL-6) [91]. MAPKs also primarily respond to PAMPs, some of which, such as SARS-CoVs, could lead to an overabundant expression of the inflammatory mediators. In contrast, it has been established that the JAK-STAT signaling pathway functions as a key mediator in IFN-β and TNF-α-induced ROS production [92,93].

**Figure 2:**
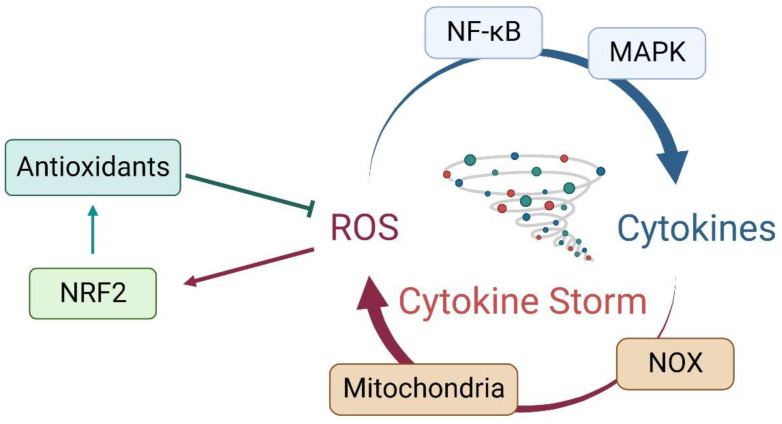
The vicious cycle between ROS and cytokines in cytokine storms. The antioxidant system negatively regulates intracellular ROS levels, which in turn affect the activity of the antioxidant system via negative feedback mechanisms to maintain redox homeostasis. Uncontrolled production of ROS may activate multiple inflammatory signaling cascades, including NF-κB and MAPK, to produce cytokines, which may in turn stimulate mitochondria and NOX family to increase ROS production. Without proper antioxidant and/or anti-inflammatory responses, the overproduced ROS and cytokines may form a positive feedback loop to amplify the inflammatory response, leading to cytokine storms.

In summary, the primary inflammatory responses and systemic inflammatory effects in cytokine storms are closely associated with overproduced ROS, which may function as DAMPs to stimulate the production, maturation, and secretion of a variety of chemokines and pro-inflammatory cytokines. In turn, the chemokines and cytokines may activate and recruit more immune cells to generate more ROS, forming an amplification loop. Without enough antioxidants and/or anti-inflammatory factors to break the ROS–cytokines vicious cycle, severe inflammation and cytokine storms would happen (Figure 2).

## 4. NRF2 in Cytokine Storms

### 4.1. NRF2 Basics

As the master transcriptional factor in the cellular adaptive antioxidant response, NRF2 regulates the genes encoding many antioxidant and phase II detoxifying enzymes and cell metabolism-related factors, including γ-glutamate-cysteine ligase catalytic and modifier subunit (GCLC and GCLM), glutathione S-transferase (GST), heme oxygenase-1 (HO-1), NAD(P)H: quinone oxidoreductase 1 (NQO1), and peroxisome proliferators-activated receptors-γ (PPAR-γ) [7,11,94]. Thus, NRF2-dependent transcription protects against oxidative damage and various redox-sensitive RCD by reducing ROS. In *Nrf2*-knockout mice, the damage to tissues and organs caused by oxidative stress is more significant than that in wild-type mice [95,96].

### 4.2. Key Cellular Events Regulated by NRF2 during Cytokine Storms

**Figure 3:**
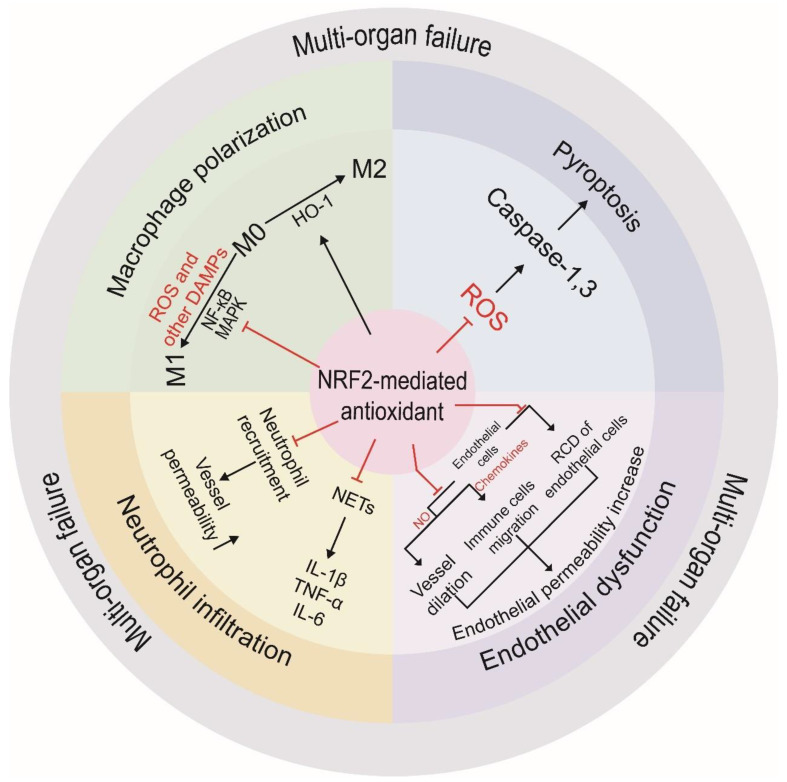
NRF2 modulates inflammatory response at different levels via multiple cellular events. (1) Macrophage polarization: NRF2-mediated antioxidant response can suppress NF-κB and MAPK to prevent macrophage polarization toward M1. In addition, NRF2-dependent induction of HO-1 can promote M2 polarization and thus promote anti-inflammatory response; (2) Pyroptosis: NRF2-mediated induction of antioxidants can decrease the accumulation of intracellular ROS and subsequently caspase-1 and 3 activation and thus pyroptosis; (3) Endothelial dysfunction: NRF2-dependent expression of antioxidant enzymes is crucial in controlling the production and secretion of NO and chemokines, and thus protecting endothelial cells from malfunction and various RCD; (4) Neutrophil infiltration: NRF2-mediated antioxidant response can suppress neutrophil recruitment and neutrophil extracellular traps (NETs) formation, resulting in reduced secretion of inflammatory cytokines.

It has been well documented that distinct cellular events, including macrophage polarization, pyroptosis, endothelial cell damage, and neutrophil infiltration, are crucial in the development of cytokine storms, in which NRF2-mediated adaptive antioxidant response can alleviate their progress, to prevent deteriorated inflammation and thus cytokine storms (Figure 3).

#### 4.2.1. Macrophage Polarization

Macrophages are complex immune cells that mainly have three phenotypes, M0, M1, and M2. Macrophage polarization, in which macrophages are converted from M0 to M1 or M2, or converted between M1 and M2, occurs in the initial stage of cytokine storms [97]. M1 macrophages are characterized by the production of high levels of pro-inflammatory cytokines, ROS, and reactive nitrogen species (RNS), as well as the induction of Type 1 helper T (Th1) responses [98]. In pathogen-induced cytokine storms, activation or polarization of innate immune cells mainly occur at the primarily infected loci, where M1 macrophages promote inflammation toward the subsequent stages of cytokine storms including multi-organ failure. In contrast, M2 macrophages are associated with anti-inflammatory responses, cell proliferation, and tissue repair [97,99].

Accumulating evidence indicates that NRF2 may function as a negative regulator of M1 polarization and a positive regulator of M2 polarization [100]. PAMPs and DAMPs, ROS in particular, are critical in the macrophage polarization toward the M1 phenotype by activating MAPK and NF-κB pathways [98]. In addition, PAMPs may also stimulate macrophages to overproduce ROS, which subsequently stimulate cytokine production. In contrast, decreased ROS levels are vital for M2 polarization [98,101].

HO-1 is a downstream target of NRF2 and is recognized as a cytoprotective enzyme via degrading heme into biliverdin, free iron, and carbon monoxide [98,102]. In sepsis models, NRF2-dependent induction of HO-1 is effective in decreasing M1 and enhancing M2 polarization by ROS scavenging and NF-κB suppression [103]. Thus, NRF2 can alleviate cytokine storms via ROS-dependent and independent mechanisms.

#### 4.2.2. Pyroptosis

Pyroptosis is a form of RCD that occurs in parenchyma cells and inflammatory cells in response to pro-inflammatory stimuli [72]. Consequently, pyroptosis-derived DAMPs may result in the activation of inflammatory cascades in the primary loci and cause systemic inflammation. Given that ROS-mediated NLRP3 is the primary activator of caspase-1, NRF2 may alleviate pyroptosis via transcriptional regulation of various antioxidants [104,105,106,107]. Similarly, the activation of caspase-3 in target cells results in extensive pyroptosis in cytokine storms induced by CAR T-cell therapy [108]. Interestingly, NRF2 can also suppress pyroptosis through caspase-3 inhibition in unilateral ureteral obstruction [109]. Furthermore, pyroptosis suppression through the NRF2 cascade has been proved to prevent secondary organ injuries induced by cytokine storms [110].

#### 4.2.3. Endothelial Dysfunction

Endothelial cells form the inner lining of the vessels and participate in the control of vascular permeability, the maintenance of blood fluidity, angiogenesis, and the trafficking of immune cells [111]. At the primary loci stage of cytokine storms, PAMPs and DAMPs released into the circulation can act on the endothelium to initiate RCD and/or alter their endocrine function to facilitate cytokine storms [2]. In turn, endothelial dysfunction contributes to systemic inflammation [112]. In virus and liposaccharide (LPS)-induced cytokine storms, NRF2 can prevent endothelial dysfunction by upregulating antioxidant enzymes, such as HO-1 and NQO-1, alleviating the death of endothelial cells [112,113]. By suppressing ROS, NRF2 can suppress a variety of RCD of endothelial cells, including ferroptosis, apoptosis, and pyroptosis [112,114,115]. PAMPs and subsequent DAMPs induce the release of NO, which mediates the relaxation of the blood vessels and the aggressive inflammatory symptoms by dilating the blood vessels [22]. Interestingly, NRF2 can effectively reduce NO production by upregulating antioxidant enzymes, including HO-1 [116].

Endothelial cells release chemokines to attract immune cells, and traffic them through the vascular wall [22,111], which further promotes endothelial permeability and induces multi-organ injuries [117]. NRF2 can suppress the recruitment of immune cells by inhibiting production of chemokines in a ROS-dependent fashion [118]. Under the action of PAMPs and/or DAMPs, endothelial cells can also produce cell adhesion molecules, including integrins, selectins, the immunoglobin superfamily, and cadherins, to recruit immune cells. NRF2 may downregulate these cell adhesion molecules by suppressing NF-κB in an antioxidant-dependent manner [113,119,120].

Endothelial cells are important in maintaining blood fluidity in normal conditions. Endothelial dysfunction is connected with coagulation disorders, including thromboembolism and disseminated intravascular coagulation (DIC) [121]. Thromboembolism and DIC are important pathological processes in cytokine storms. Coagulation disorders can contribute to vascular occlusion, hemorrhage, and tissue necrosis [2,121,122]. Endothelial dysfunction may cause the antithrombotic surface to be destroyed and coagulator leakage, which will finally cause a coagulation disorder [123]. These processes are regulated by ROS, and NRF2 can suppress ROS to alleviate these coagulation disorders [112].

#### 4.2.4. Neutrophil Infiltration

In cytokine storms, neutrophils are the most important immune cells recruited by chemokines and cell adhesion molecules, leading to multi-organ injuries and increased endothelial permeability. Neutrophils also form NET, a three-dimensional web-like structure composed of DNA, histones, and granular proteins, to elicit an innate immune response by entrapping microorganisms [117,124]. In addition, NETs may amplify cytokine production in the primary response and exacerbate secondary injuries during cytokine storms [117]. Accumulating evidence indicates that NRF2 plays vital roles in regulating neutrophil recruitment in an antioxidants-dependent fashion [124,125,126]. By suppressing ROS, NRF2-dependent transcription can promote rolling velocity and reduce the adhesion to inhibit neutrophil recruitment [119,126].

Several studies have shown that NRF2 activators can decrease neutrophil infiltration and cytokine levels, including IL-1β, TNF-α, and IL-6, leading to the failure of NETs [119,124]. In addition, NRF2-mediated transcription of antioxidant enzymes reduces ROS levels and suppresses neutrophil production of IL-6 and TNF-α in sepsis models [127,128]. Furthermore, NRF2 has also been known to upregulate antioxidant enzymes in healthy neutrophils and reduce ROS, and alleviate the production of IL-6 and TNF-α in neutrophils from septic patients [129].

In summary, the NRF2-mediated antioxidant response can alleviate inflammatory responses via regulating many crucial cellular events during cytokine storms, including macrophage polarization, pyroptosis, endothelial cell damage, and neutrophil infiltration (Figure 3). It is of worth noting that the molecular mechanisms underlying the regulatory roles of NRF2 in the key cellular events during cytokine storms are complicated and warrant further detailed investigations.

### 4.3. NRF2 Regulates the Transcription and Maturation of Cytokines

**Figure 4:**
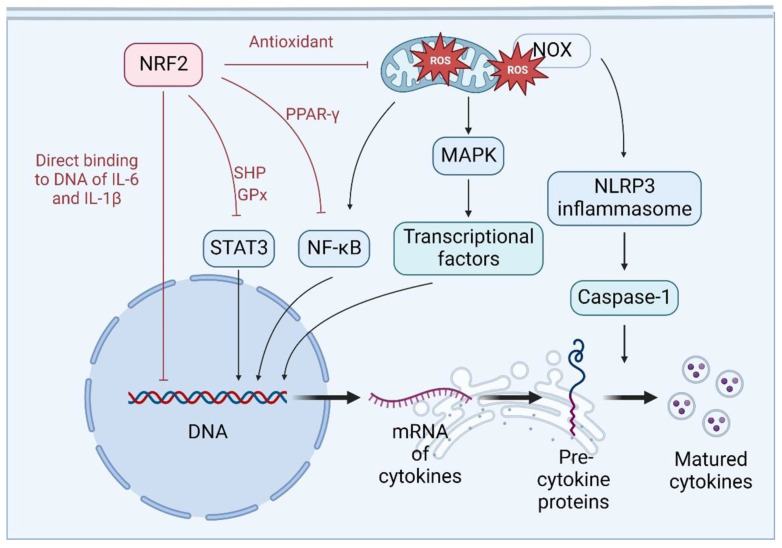
Molecular details underlying NRF2-mediated downregulation of cytokines. The cytokine production process includes transcription, translation, and maturation. NF-κB, STAT3, and MAPK-activated transcription factors can promote the transcriptional process. By induction of antioxidant enzymes, NRF2 can suppress NF-κB and MAPK in an ROS-dependent manner. In addition, NRF2 can also inhibit the activation of NF-κB and STAT3 via induction of PPAR-γ and small heterodimer protein (SHP), respectively. In addition, NRF2 can suppress NLRP3 inflammasome and thus IL-1β maturation in an antioxidant-dependent mechanism.

In cytokine storms, the overexpression of cytokines is the most noticeable feature because they are the primary molecules connecting all the stages. Interestingly, NRF2 may modulate cytokine production and release, not only at the primary loci but also the systemic levels, in which the transcription and maturation of cytokines are sensitive to ROS accumulation [110,130]. As illustrated in Figure 4, NRF2 activation, on the one hand, downregulates the mRNA levels of pro-inflammatory cytokines, including TNF-α, IL-6, and IL-1β, by inhibiting NF-κB activation [100,131,132]. In line with this concept, in the absence of NRF2, the mRNA levels of pro-inflammatory cytokines are much higher than those in wild-type conditions [12]. On the other hand, NRF2 positively affects the transcription of anti-inflammatory cytokines, such as IL-10 [133,134,135]. The mechanisms for NRF2 downregulating cytokine levels at the transcriptional level are complex. First, NRF2 can regulate cytokine transcription by acting directly on the DNA, in which NRF2 binds to the promoters of cytokine genes, including IL-6 and IL-1β, reducing their transcription [10,13]. Second, NRF2 can block the recruitment of RNA polymerase II (RNA Pol II), thereby suppressing the transcription process [10]. In contrast, it has also been demonstrated that NRF2 plays a positive role in IL-6 transcription by binding directly to the promoter [136,137], suggesting that NRF2 is critical in the fine tuning of IL-6 expression under complicated inflammatory conditions.

Cytokines are directly regulated by multiple transcription factors and posttranslational mechanisms, including NF-κB, MAPKs, and STAT. The activity of NF-κB can be affected by ROS, which are tightly controlled by antioxidant systems [88,89]. By reducing phosphorylation of IκBα, NRF2-dependent antioxidants can suppress NF-κB activation [138,139]. The phosphorylation of IκBα is catalyzed by IKKβ, which may also be activated by ROS. In addition, NRF2 suppresses NF-κB by upregulating downstream antioxidant enzymes, including superoxide dismutase (SOD), HO-1, and NQO1 [100,140,141]. In cytokine storms caused by sepsis and IAV infections, NRF2 activation results in reduced availability of ROS, NF-κB inhibition, and decreased cytokine production [142,143]. In addition, the mRNA and protein levels of NF-κB are downregulated by NRF2 activation in an antioxidant-dependent way [138,139].

Activation of MAPKs may also be suppressed by NRF2 activation [144]. ROS are capable of initiating MAPK cascades, in which each layer of kinase phosphorylates the lower layer and finally activates MAPK [145]. As a protein kinase, MAPK further activates transcription factors to increase the transcription of various cytokine genes [146]. In cytokine storms derived from sepsis or IAV infections, an NRF2-dependent antioxidant response reduces the phosphorylation of p38, ERK, and c-Jun N-terminal kinase (JNK), all of which are members of the MAPK family, and further suppresses cytokine production, including IL-1β, IL-6, TNF-α, and IL-8 [143,147,148]. In an inflammation model induced by ischemia–reperfusion, NRF2 showed anti-inflammatory effects by upregulating HO-1 and suppressing p38 in an antioxidant-dependent manner [149]. Thus, NRF2 may suppress MAPK-mediated production of cytokines by reducing ROS levels during cytokine storms.

Previous studies, including our own, have shown that NRF2 promoted PPAR-γ transcription by directly binding to its promoter in adipocytes and hepatocytes [150,151,152]. In acute pulmonary inflammation, NRF2 activates PPAR-γ but suppresses the activity of NF-κB [153]. In line with these findings, PPAR-γ has also been found to suppress sepsis-induced cardiac cell injury, a common secondary organ injury in cytokine storms, by suppressing NF-κB and MAPKs [154]. With regard to the molecular details on the crosstalk between PPAR-γ and NF-κB, PPAR-γ has been found to suppress NF-κB activity via binding directly to NF-κB and/or inhibiting its co-activators [155,156]. In addition, PPAR-γ may suppress the activation of NF-κB and MAPKs by suppressing ROS production [155,157]. Hence, it is reasonable to speculate that NRF2 may mitigate cytokine storms by activating PPAR-γ. Nevertheless, the mechanisms regarding the mitigating effects of NRF2-mediated PPAR-γ activation on cytokine storms still need further investigation.

In agreement with the consensus that NRF2 negatively affects NF-κB activation, KEAP1 has also been found to be involved in the regulation of NF-κB activity [158]. Our previous studies showed that KEAP1 silencing results in exaggerated NF-κB activation and augmented inflammatory responses in LPS-challenged macrophages [137]. With regard to the mechanisms underlying the regulation of KEAP1 on NF-kB, Lee et al. reported that KEAP1 directly binds to IKKβ, which is important in the activation of NF-κB, leading to ubiquitin–proteasome degradation of IKKβ and subsequent inhibition of NF-κB [159]. In addition, KEAP1 has also been found to enhance the degradation of IKKβ through the autophagy–lysosome pathway [160]. As NRF2 can positively regulate KEAP1 [161], it is reasonable to speculate that NRF2 suppresses NF-κB by upregulating KEAP1. However, the regulatory function of the NRF2–KEAP1 feedback loop on NF-κB in cytokine storms still lacks direct experimental evidence.

STAT3 is a transcriptional factor that can promote the transcription of cytokine genes, and therefore plays a crucial role in cytokine storms. NRF2 suppresses the STAT3 pathway in two different modes. Firstly, NRF2 can regulate STAT3 via an antioxidant-independent mechanism, in which NRF2 promotes the expression of SHP. SHP is a nuclear receptor that not only binds to phosphorylated STAT3 to suppress its activity [162], but also regulates TLR- and NLRP3-induced inflammasome activation [163]. Secondly, NRF2 suppresses STAT3 activation by inhibiting its phosphorylation, whereby NRF2 induces glutathione peroxidase 2 (GPx2) to suppress JAK1, a key kinase for STAT3 phosphorylation [164]. It is worth noting that there is scarce direct experimental evidence supporting the idea that NRF2 can suppress STAT3 to alleviate cytokine storms.

In addition to regulating the transcription of cytokines, NRF2 may also be involved in the maturation of cytokine proteins by inhibiting the activation of key proteins via ROS-dependent mechanisms. Inflammasomes participate in cytokine maturation by promoting the development of caspases, which can cleave IL-1β and IL-18 precursors into bioactive forms [77,165]. While NRF2 activation is associated with the decrease of NLRP3 and caspase-1 [110,166,167], the maturation of IL-1β is closely associated with the presence of caspase-1. NLRP3 inflammasome, which can be activated by ROS, plays a crucial role in processing the precursor of caspase-1 into its mature form [77,165]. NRF2 suppresses the LPS-induced activation of the NLRP3 inflammasome by ROS scavenging, further decreasing the production of caspase-1 and IL-1β [105]. In sepsis-induced cytokine storms, NRF2 initiates an anti-inflammatory process by suppressing inflammasome and caspase-1 and alleviating mitochondria dysfunction [167]. The same phenomenon is also observed in a sepsis-induced liver injury model [110]. Of note, the antioxidant enzymes HO-1, SOD, and NQO1 were elevated in both experiments, indicating that NRF2 suppresses protein modifications in an antioxidant-dependent way.

In summary, the expression and maturation of a variety of cytokines are tightly regulated by ROS-mediated or redox-sensitive mechanisms, in which NRF2 plays crucial roles in antioxidant-dependent and independent manners (Figure 4).

### 4.4. Strategies to Mitigate Cytokine Storms by Targeting NRF2

Emerging evidence suggests that NRF2 is a valuable target for the prevention and treatment of cytokine storms. Biopsies from COVID-19 patients showed that genes associated with the NRF2-dependent antioxidant response, such as NQO1, PPAR-γ and HO-1, were decreased [27,168,169], suggesting that NRF2 activation could be a therapeutic strategy for COVID-19. Many proven NRF2 activators, including dimethyl fumarate (DMF), sulforaphane (SFN), curcumin, and PB125^®^, have been applied in treating inflammation-related disorders [170]. In septic mice, DMF treatment can downregulate the levels of TNF-α and IL-6 in the brain [171]. An in vitro experiment proved that DMF could suppress the SARS-CoV-19-induced inflammatory response [168]. Pretreatment of septic mice with SFN decreased the levels of TNF-α and IL-6 in lung and brain tissues [119,172]. In addition, SFN treatment increased the survival rate of mice, and alleviates the multi-organ injury induced by sepsis, including lung, liver, and kidney [173,174]. Curcumin also showed a protective effect against IAV infection-induced lung injury by reducing the levels of TNF-α, IL-1β, and IL-6 in the tissues [147,175]. Recently, a clinical trial showed that nano-curcumin could combat cytokine storms in COVID-19 patients [176]. In addition to those well-documented NRF2 activators, some novel phytochemicals can activate NRF2 to alleviate inflammation. In bile duct ligation mice, pentoxifylline was reported to suppress inflammation by activating NRF2, and prevent liver cirrhosis [177]. Vincamine protects mice from LPS-induced lung injury by activating NRF2 [178]. Hydroalcoholic curry leaf extract has been found to suppress IL-1β, IL-6, and TNF-α, and activate NRF2 in acute pancreatitis mice [179]. Although more clinical trials are required, studies have clearly shown that NRF2 is a valuable target in mitigating cytokine storms.

As detailed above, NRF2 modulates cytokine levels mainly through antioxidant-dependent indirect ways, in which ROS serve as key mediators. Given that ROS and the ROS-sensitive redox microenvironment are crucial for the activation of multiple cytokine-producing signal cascades, including NF-κB and MAPK, ROS and oxidative stress have been recognized as key targets to control inflammation. However, ROS function as a double-edged sword in inflammation because they are needed by the immune system to remove pathogens. In addition, ROS may also function as signaling molecules, mediating many critical physiological processes and stress responses [180,181,182]. Thus, NRF2-mediated induction of antioxidants, on the one hand, may suppress ROS to alleviate inflammation-related tissue injuries. On the other hand, sustained NRF2 activation may lower the availability of physiological ROS, resulting in weakened antimicrobial abilities of macrophages and neutrophils and impaired redox signaling. In consideration of the paradoxical roles of ROS and NRF2-mediated adaptive responses in inflammatory responses, it is important to determine when, where, and how to modulate NRF2 to effectively mitigate inflammation and cytokine storms and avoid any potential off-target effects.

Cytokine storms result from complex interactions of a variety of cells and systems, in which the roles of activated NRF2 and its crosstalk with diverse signaling pathways are not entirely defined. For example, it is still unclear whether NRF2 activation is involved in immunoparalysis, a period or state of immunosuppression following an initial hyperinflammatory response in sepsis. Because immunoparalysis is related to a high risk of sepsis and organ failures, the application of NRF2 activators to downregulate the immune system should be performed under constant surveillance to prevent exacerbations of infections. When facing immunoparalysis in particular, the NRF2 activation strategy should be considered with more caution. At the early stage of the acute response phase, proper activation of NRF2 could be considered an effective anti-inflammatory strategy. In contrast, the effectiveness of NRF2 activators in later stages is in doubt, because NRF2 has also been shown to have various pro-inflammatory effects [183].

In summary, the spatiotemporal roles of NRF2 in cytokine storms still need more detailed research to support the development of new intervention strategies. In addition, novel NRF2 activators with high activity and specificity are required to facilitate their potential applications in treating cytokine storms. Clearly, early surveillance and corresponding measures are needed to improve the prognosis of patients when NRF2 modulators are used to prevent and/or treat cytokine storms.

## 5. Conclusions and Perspectives

NRF2 is critical in regulating the fundamental cellular, transcriptional, and maturation events of cytokine storms. Thus, it is reasonable to consider NRF2 as a potential therapeutic target for various inflammation-related disorders. The challenges for controlling cytokine storms lie in dissecting the exact physiological and pathophysiological regulations of the inflammatory response. Expanding knowledge on NRF2-ARE signaling during inflammation would advance our understanding of cytokine storms and reveal potential applications in drug discovery. Given that ROS and NRF2 play a dual Yin–Yang role in a wide range of physiological and pathophysiological processes, NRF2, as the master regulator in charge of transcriptional control of many key antioxidant enzymes, may play paradoxical roles in redox signaling and cellular homeostasis. Depending on the nature of pathophysiological conditions, NRF2 could be the good, the bad, and the ugly. Suggested areas for future study include the molecular mechanisms of cytokine production and release and the spatiotemporal roles of NRF2 in the context of cytokine storms, including therapeutic settings. We anticipate that significant advances will be made in the near future to expand our understanding of NRF2 signaling pathways and to develop new drugs for cytokine storm-related diseases.

## Data Availability

The data presented in this study are available in the article.

## Abbreviations

ARE: antioxidant response element; bZIP: basic region-leucine zipper; CAR: chi-meric antigen receptor; COVID-19: coronavirus disease-19; Cul3: Cullin 3; DAMPs: damage-associated molecular patterns; DIC: disseminated intravascular coagulation; DMF: dimethyl fumarate; ERK: extracellular regulated protein kinases; GCLC: glutamate–cysteine ligase catalytic subunit; GCLM: glutamate–cysteine ligase modifier subunit; GPx2: glutathione peroxidase 2; GST: glutathione S-transferase; HO-1: Heme oxygenase-1; IAV: influenza A virus; IFN: interferon; IL: interleukin; IκB: inhibitor of NF-κB; IKK: IκB kinase; JAK-STAT: Janus kinase/signal transducer and activators of transcription; JNK: c-Jun N-terminal kinase; KEAP1: Kelch-like ECH-associated protein 1; LPS: lipopolysaccharide; MAPK: mitogen-activated protein kinase; mIL-6R: membrane-bound IL-6 receptor; NETs: neutrophil extracellular traps; NF-κB: nuclear factor kappa-light chain-enhancer of activated B; NK: nature kill; NLRP3: nucleotide-binding domain, leucine-rich-repeat-containing family, pyrin domain-containing 3; NOD2: nucleotide-binding oligomerization domain-containing protein 2; NOX: NADPH oxidase; NQO1: NAD(P)H: quinone oxidoreductase 1; NRF2: nuclear factor erythroid 2-related factor 2; PAMPs: pathogen-associated molecular patterns; PPAR-γ: peroxisome proliferators-activated receptor-γ; PRRs: pattern recognition receptors; RCD: regulated cell death; RNS: reactive nitrogen species; ROS: reactive oxygen species; SARS-CoV-2: severe acute respiratory syndrome coronavirus 2; SFN: sulforaphane; SHP: small heterodimer protein; SOD: superoxide dismutase; TLRs: Toll-like receptors; TNF-α: tumor necrosis factor α; Th1: Type 1 helper T.
